# Regulation of serine protease inhibitor Kazal type-5 (SPINK5) gene expression in the keratinocytes

**DOI:** 10.1007/s12199-014-0393-7

**Published:** 2014-06-04

**Authors:** Ngoc Anh Le, Midori Katsuyama, Masashi Demura, Hideji Tanii, Hironobu Katsuyama, Kiyofumi Saijoh

**Affiliations:** 1 Department of Hygiene, Kanazawa University School of Medicine, Kanazawa, 9208640 Japan; 2 Department of Public Health, Kawasaki Medical University, Kurashiki, 7011092 Japan

**Keywords:** Serine protease inhibitor Kazal type-5 (SPINK5), Human keratinocyte, GATA3, Transcription regulation

## Abstract

**Objectives:**

Serine protease inhibitor Kazal type-5 (SPINK5) plays a crucial role in deciding the timing of desquamation of the skin. Its gene expression is limited at the very surface of the stratum granulosum (SG), whereas expression of kallikreins (KLKs) encoding proteases is usually found throughout the stratum spinosum and SG.

**Methods:**

To explore the difference in expression regulation of these proteases/inhibitors, the function of SPINK5 promoter was examined using luciferase assay.

**Results:**

Luciferase assay targeting the SPINK5 promoters (nucleotide −676/−532 and −318/−146 from the major transcription start site) showed high intensity in NHEK human keratinocyte. These two sites had neither common *cis*-elements nor GATA3 element but electrophoretic mobility shift assay showed similar retardation bands. Moreover, DNA footprinting did not display specific protected bands. Thus, we could not identify *cis*-element(s) that controlled these elements. Differentiation induced by high Ca^2+^ medium failed to alter their luciferase activities. Transfection of GATA3 expressing vector significantly but slightly increased them and that of vector expressing its dominant negative form decreased.

**Conclusions:**

Although GATA3 is reportedly important for inhibition of proliferation and induction of differentiation of keratinocytes, its effect on SPINK5 expression was indirect and GATA3 alone was insufficient for final differentiation of keratinocytes where full SPINK5 expression was observed.

## Introduction

To maintain a steady number of the stratum corneum (SC) layers is an important factor for the solid skin barrier function. In the SC adjacent to the stratum granulosum (SG), corneocytes are tightly bound to each other through the corneodesmosomes consisted of extracellular corneodesmosin, transmembrane desmoglein-1 and desmocollin-1, and cytoplasmic desmosomal plaque proteins (plakoglobin) [1, 2]. These firm contacts reduce toward the surface and finally disappear on the surface where desquamation of the corneocytes occurs [3]. This reduction is due to corneodesmosome degradation caused by serine protease activities supplied by the tissue kallikrein gene (KLK) family proteins consisting of fifteen members known as KLK1 to KLK15 [4]. Their activities are regulated by serine protease inhibitor Kazal type-5 (SPINK5) [5, 6] to match the shedding schedule to the proliferation rate of keratinocytes [7, 8]. This turnover is very steady and maintained regardless of age [9, 10] on behalf of serine protease-SPINK5 balance. In fact, the loss of function mutation of SPINK5 gene causes hyperdesquamation and loss of the SC known as Netherton syndrome [11–13].

Thus, KLK and SPINK5 proteins co-localize and interact with each other at the SC [14]. Nonetheless, expression of KLKs is usually found throughout the stratum spinosum (SS) and SG [5] and, on the other hand, expression of SPINK5 is limited at the very surface of SG [12]. GATA3 plays a very important role for the ubiquitous expression of KLKs [15], whereas the major regulators of local expression of SPINK5 are yet known. Limited expression of SPINK5 plays a crucial role on avoidance of premature- and/or hyperdesquamation. To clarify the basis of such limited expression of SPINK5, the function of its promoter was explored in the present study.

## Materials and methods

### Keratinocyte culture

Normal human keratinocyte (NHEK; Kurabo, Osaka, Japan, several different single donors) was inoculated at a concentration of 2,500 cells/cm^2^ in culture media, HuMedia-KG2 (Kurabo) supplemented with insulin 10 µg/ml, human epidermal growth factor 0.1 ng/ml, hydrocortisone 0.5 µg/ml, bovine pituitary extract (BPE) 0.4 % v/v, gentamycin 50 µg/ml and amphotericin B 50 ng/ml. The culture was maintained at 37 °C in a 95 % O_2_/5 % CO_2_ humidified chamber. The cells were subcultured twice in the media of which calcium concentration was as low as 0.15 mM to avoid differentiation. In vivo differentiation was induced as described elsewhere [16]. In brief, at the second passage when it became 40–60 % confluent, the media was changed to normal Ca^2+^ medium (0.15 mM Ca^2+^; low Ca^2+^) or high Ca^2+^ medium (1.5 mM Ca^2+^) in the absence of BPE and cultured for 2 days.

### Reporter vectors

The proximal promoter of the human SPINK5 gene [nucleotide −1 to −1141 from the major transcriptional start site (Accession No; Chromosome 5—NC_000005.9)] was PCR amplified using Sac I- and Nhe I-tailed primers, respectively, −1141 and −1 (Table 1), from the genome of NHEK as a template. Evolutional conservation search between human and Rhesus macaque (rheMac2) using ECR browser (http://ecrbrowser.dcode.org) displayed that −1 to −1214 was a putative promoter enhancer. Unfortunately, the primer search program (DNasis-Mac v3.6) selected a primer start with −1141 instead of −1214 was suitable for PCR amplification. After digestion with Sac I and Nhe I, the amplified products were initially cloned into pGL4.10[luc2] (Promega, Madison, WI, USA), designated as −1141/−1. To generate clones with different 5′ terminal length, PCR was performed using different 5′ primers for deletion and the 3′ primer to conserve pGL4 sequence (Table 1). After digestion with Sac I, the products were re-ligated to obtain deletion clones. All cloned promoters in the reporter construct were sequenced to confirm that their sequence was conserved after cloning.

**Table 1:** Primers to obtain SPINK5 promoter

For initial clone		Downstream primer	Promoter included
−1141	SacI-CTGGAATGAAACAATTTGGCTCTGG	−1	−1141/−1
−1	NheI-TGCTCAGCTGGTGCAGTATGACT		
For deletion			
−1076	SacI-GCTATTAGGGCAAAATAAGCTGGTA	pGL4	−1076/−1
−932	SacI-GAGACTAAATAATGGGATGATACAGG	pGL4	−932/−1
−861	SacI-CGGTAATATGGGTAATCATAGGCTA	pGL4	−861/−1
−676	SacI- GGATGAAGCAGTGTACAGAGAGG	pGL4	−676/−1
−555	SacI-AGTTCCTGCCTCCCTATATATTCA	pGL4	−555/−1
−437	SacI-CCCTGGATTTCTCTGGATGTGGA	pGL4	−437/−1
−318	SacI-GTCTACGAGGTAGTATATCTGGTAT	pGL4	−318/−1
−169	SacI-ATTCTCTGTTCAAACCTGACACCC	pGL4	−169/−1
pGL4	Sac I-AGGCCAGAGAAATGTTCTGGCACC		
For fragmentation			
−555c	TGAATATATAGGGAGGCAGGAACT	−677	−676/−532
−169c	GGGTGTCAGGTTTGAACAGAGAAT	−318	−318/−146

Numbers indicate the location of 5′ terminal base counted from the transcription initiation site. Primers −555c and −169c were complementary to −555, and −169, respectively, and hence the numbers were the locations of 3′ terminal base

### Other vectors used

pGL−954/+40; the vector possessed the highest luciferase activity among reporter vectors containing KLK1 promoter [15], pcDNA3.1 (Invitrogen, Carlsbad, CA, USA), pcGATA3; the coding region of GATA3 (the open reading frame with 119 bp upstream to the start codon and 33 bp downstream from the stop codon) was cloned into pcDNA3.1, and pcGATA3mut; to obtain dominant negative mutant of pcGATA3, its sequence corresponding to 305KRR was changed to AAA [15].

### SPINK5 promoter activity in NHEK cells

NHEK cells cultured on 24-well plates were transfected with 0.75 μg of reporter vectors pGL4.10[luc2] cloned with different fragments from the proximal promoter of the human SPINK5 using Tfx™-20 (Promega, Madison, WI, USA). After 48 h, the cells were lysed using a luciferase reporter assay system (Promega, Madison, WI, USA) and the firefly luciferase activities were measured with a Lumat LB9507 (Berthold Technologies, Tokyo, Japan). All the experiments were performed at least three times for each reporter plasmid and the relative luciferase activity was calculated. Results were presented as the mean ± SD. To confirm that the putative sequence was really active, −676/−532 or −318/−146 was PCR amplified using a set of primers (Table 1), used for electrophoretic mobility shift assay (EMSA) and cloned into pGL4.10[luc2] for the luciferase assay.

### Search for putative cis-elements

To find putative *cis*-elements, sequence of −1141/−1 was subjected to TFSEARCH (http://www.cbrc.jp/research/db/TFSEARCH.html), ALGGEN PROMO [17, 18] and PhysBinder (http://bioit.dmbr.ugent.be/physbinder/ [19]).

### Electrophoretic mobility shift assay (EMSA)

According to the results of luciferase assay, −676/−532, and −318/−146 supposed to contain activating element, were PCR amplified using primers listed in Table 1. Nuclear protein was obtained from 1 × 10^6^ NHEK cells cultured with KG2, or 0.15 and 1.5 mM Ca^2+^ media without BPE, based on the protocol [20]. In brief, after washing with ice-cold PBS (Ca^2+^ and Mg^2+^ free), cells were resuspended with 5 volumes of ice-cold cell homogenization buffer (10 mM HEPES–KOH, pH7.8, 1.5 mM MgCl_2_, 10 mMKCl, 0.5 mM DTT, 0.5 mM PMSF, and 1 × proteinase inhibitor cocktail (Nakalai tesque, Kyoto, Japan) to stand on ice for 10 min, and collected by centrifuging at 250*g* for 10 min. The cell pellet was resuspended in 3 volumes of ice-cold cell homogenization buffer containing 0.05 % (v/v) Nonidet P-40, and homogenized on ice with 20 strokes of a tight-fitting Dounce homogenizer. The nuclei were collected by centrifugation at 250*g* for 10 min at 4 °C. After removing the supernatant, the nuclei pellet was resuspended with 20 ml of cell suspension buffer (40 mM HEPES–KOH, pH 7.8, 0.4 M KCl, 10 % glycerol, 1 mM DTT, 0.1 mM PMSF, and 1 × proteinase inhibitor cocktail). NaCl was added to a final concentration of 300 mM, and the resuspended nuclei were incubated on ice for 30 min then centrifuged at 15,093*g* at 4 °C for 15 min. The supernatant containing nuclear protein was divided into aliquots and stored at −80 °C until use. Protein concentration was determined by the Bradford method on Model 680 Microplate Reader (Bio-Rad, Hercules, CA, USA). After 100 ng of each DNA was mixed with 6 μg of nuclear protein in a final volume of 20 μl of binding buffer containing 10 mM Tris/HCl (pH 8.0), 50 mM KCl, 3.5 mM DTT, 0.25 % Tween 20, and 0.025 μg salmon sperm DNA and allowed to stand for 25 min at room temperature, electrophoretic mobility shift assay (EMSA) was performed on 1 % agarose/ethidium bromide gel. Poly(dI-dC) as DNA and BSA as protein were served as negative control.

### DNA footprinting

To clarify the location that nuclear protein was attached, DNA footprinting using DNase I protection was performed on pGLSP−676/−532 and pGLSP−318/−146 that showed high luciferase activities. pGL−954/+40 was served as positive control. These DNAs were bound with nuclear protein as described above. To this mixture, 1 U RQ1 RNase-free DNase I (Promega) and supplied DNase I buffer were added and allowed to stand at room temperature for 1 min. The digested DNAs were phenol/chloroform extracted and ethanol precipitated. Footprinting ladder was PCR synthesized using PCR primer, RVprimer3 by BigDye (Applied Biosystems, CA). The sequence was analyzed by ABI Prism 3100 (Applied Biosystems).

### Statistical analysis

To clarify statistical significance among luciferase activities, one-way ANOVA with Tukey’s HSD test as post hoc test was used.

## Results

**Fig. 1:**
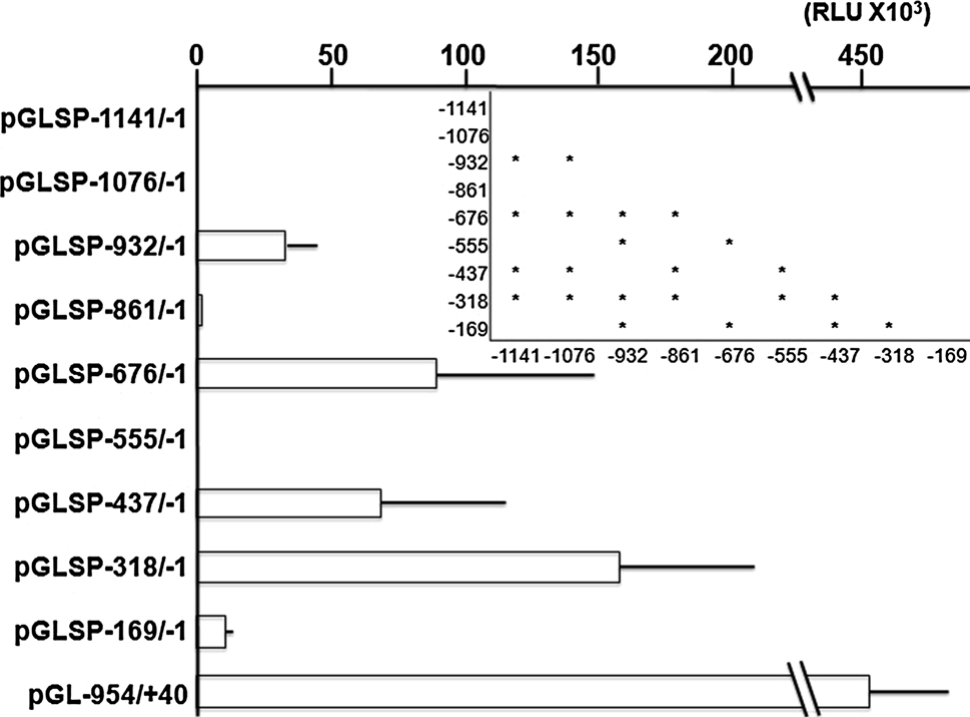
Relative luciferase activities of reporter plasmids containing different lengths of putative SPINK5 promoter in NHEK. Each column represents the mean of three independent experiments, each done in triplicate; *bars*, ± SD. *Inset*; Significant differences in the intensity between the groups (*P* < 0.05 One-way ANOVA and post hoc test using Tukey’s HSD test). When pGL−954/+40 was transfected, the intensity was higher than 450 × 10^3^ RLU

To examine the location of putative activating and/or inhibitory *cis*-elements, reporter vectors were transfected into NHEK cells and luciferase assay was performed. Transcriptional activity was almost null when pGLSP−1141/−1 and pGLSP−1076/−1 were transfected to NHEK (Fig. 1). When pGLSP−932/−1, a clone deleted −1141 to −932, was transfected, a significantly higher intensity than pGLSP−1141/−1 and pGLSP−1076/−1 was observed. Moreover, transfection of pGLSP−676/−1, a clone deleted −1141 to −676, and pGLSP−318/−1, a clone deleted −1141 to −318, gave significantly higher intensity than other clones. The intensities of these two clones did not show significant difference. The intensity of pGL−954/+40 containing KLK1 promoter was more than three times higher than that of pGLSP−318/−1.

### Effect of high Ca^2+^ medium on transcriptional activity of SPINK5 putative promoter

**Fig. 2:**
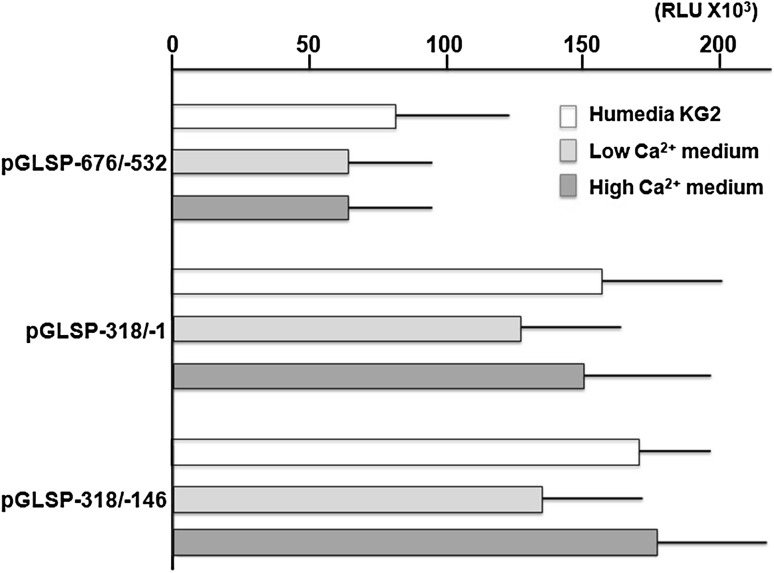
Effect of high Ca^2+^ medium on relative luciferase activities of SPINK5 putative promoters. pGLSP−676/−532, pGLSP−318/−1 and pGLSP−318/−146 were transfected to normal HuMedia-KG2-, low Ca^2+^ medium-, and high Ca^2+^ medium-cultured NHEK. Each column represents the mean of three independent experiments, each done in triplicate; *bars*, ± SD. No significant difference was observed in any of pGLSP−932/−1, pGLSP−676/−1 and pGLSP−318/−1 among different culture media (One-way ANOVA and post hoc test using Tukey’s HSD test)

Intensities of pGLSP−676/−1 and pGLSP−318/−1 were significantly higher than that of pGLSP−932/−1, and those of pGLSP−318/−1 and pGLSP−318/−146 were almost the same (Fig. 2). Thus, pGLSP−676/−1, pGLSP−318/−1 and pGLSP−318/−146 were subjected to examine the effects of high Ca^2+^ medium. Removal of BPE from Humedia-KG showed no significant differences in the intensities of pGLSP−676/−532, pGLSP−318/−1 and pGLSP−318/−146 (Fig. 2). High Ca^2+^ media-cultured NHEK also did not show any differences in their intensities.

### TFSEARCH, ALGGEN PROMO and PhysBinder to search putative *cis*-elements

Sequence of −1141/−1 was subjected to TFSEARCH, ALGGEN PROMO and PhysBinder for searching putative *cis-*elements. Among elements hit by these programs, those that did not exist in −1141/−933, −860/−677, −554/−319, −169/−1 were listed (Table 2). Excluding SRY, no common *cis*-elements existed in the activating regions −932/−837, −676/−532 and −318/−146. A point mutation on −206 G to A reportedly produces GATA3-binding site [21], but our −206 was G. Thus, no GATA3-binding site was extracted in any of the activating regions.

**Table 2:** Putative *cis*-elements located at −932/−837, −676/−532, and −318/−146

−932/−862	−676/−532	−318/−146	
POU2F2	STAT5A	HNF-4a	HSF1
SRY	AML-1a	LEF-1	HSF2
E2F	Nkx-2	TCF-4	Oct-1
YY-1		SRY	CDP-CR
		TCF-4E	MZF-1
		STAT1	

Putative *cis*-elements were searched using TFSEARCH (www.cbrc.jp/research/db/TFSEARCH.html) and ALGGEN PROMO [17, 18] and PhysBinder [19]. Those did not exist in −1141/−933, −860/−677, −554/−319, −169/−1 were listed

### Electrophoretic mobility shift assay (EMSA)

**Fig. 3:**
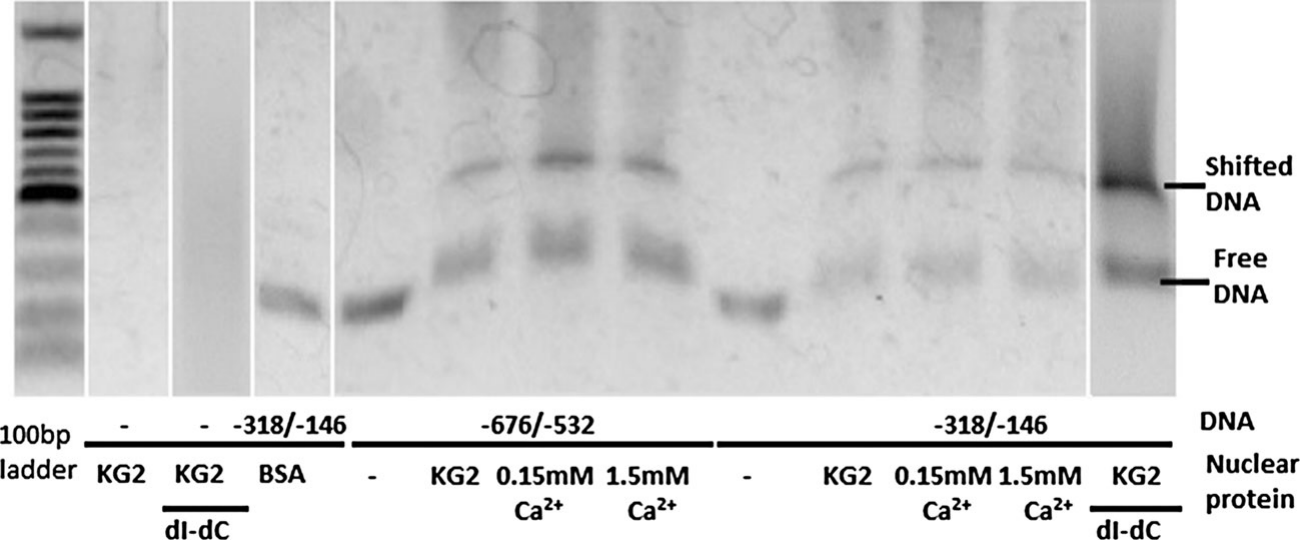
EMSA for fragments −676/−532 and −318/−146. To avoid non-specific binding, poly(dI-dC) was utilized. Aside from nuclear proteins, bovine serum albumin (BSA) was served as negative control

−676/−532 and −318/−146 supposed to contain activating element were PCR amplified using primers listed in Table 1 and subjected to EMSA (Fig. 3). Mobility of both fragments was delayed by all of nuclear extracts from normal HuMedia-KG-, low Ca^2+^ medium-, and high Ca^2+^ medium-cultured NHEK. Location of retarded bands was almost identical regardless of −676/−532 or −318/−146 and regardless of culture media.

### Effect of pcGATA3 and pcGATA3mut on SPINK5 promoter

**Fig. 4:**
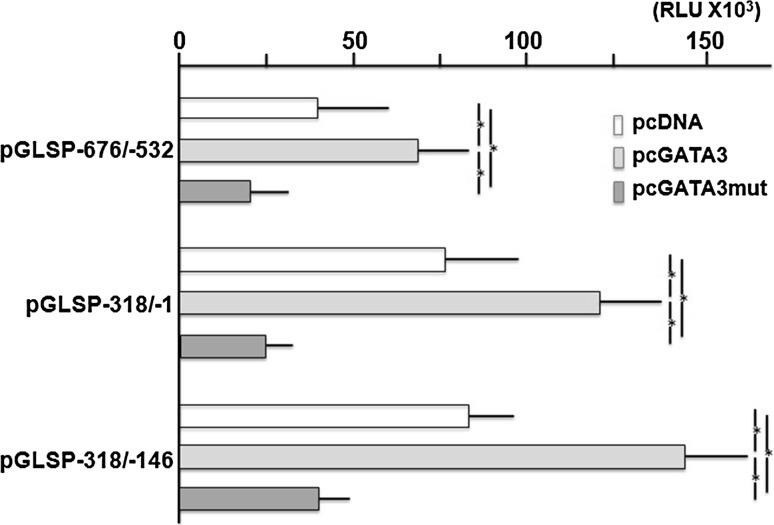
Effects of co-transfection of pcGATA3 and pcGATA3mut with pGLSP−676/−532, pGLSP−318/−1 and pGLSP−318/−146. Each column represents the mean of three independent experiments, each done in triplicate; *bars*, ± SD. *Significant difference (One-way ANOVA and post hoc test using Tukey’s HSD test)

Co-transfection of pcGATA3 with pGLSP−676/−532, −318/−146 and −318/−1 increased the intensities significantly (Fig. 4). However, their intensities were still <1/3 of that of pGL−954/+40. On the other hand, pcGATA3mut co-transfection decreased the activities significantly in any of the plasmids.

### DNA foot printing

**Fig. 5:**
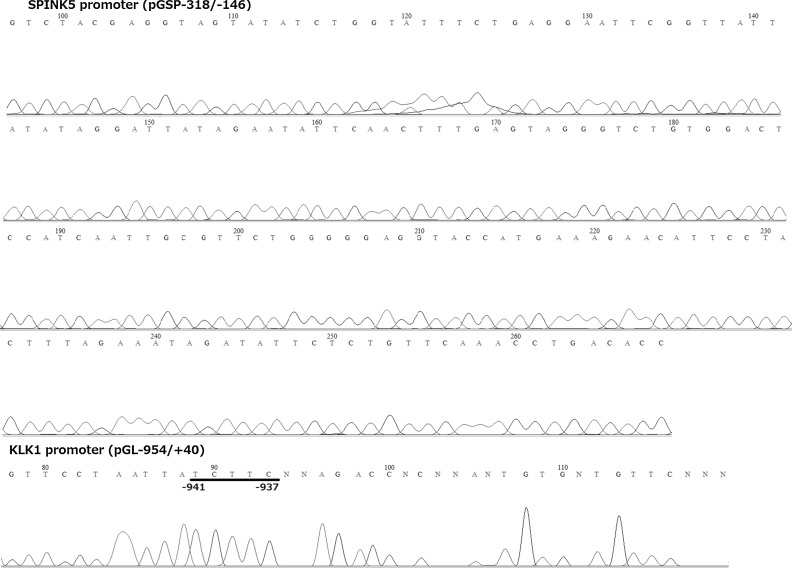
Sequence ladder of pGSP−318/−146 (Upper) and pGL−954/+40 (Lower; positive control) after digestion by DNaseI along with nuclear protein. Sequence ladder was made by the primer, RV3 (supplied for pGL4.10[luc2]). *Underline*; protected sequence where TFSEARCH, ALGGEN PROMO, and PhysBinder selected STAT4, c-Ets-1, c-Ets-2, Elk-1, Pax-5 and p53 as putative *cis*-elements

When pGLSP−676/−532 and pGLSP−318/−146 were subjected to DNA footprinting, no specific sequence was protected (pGLSP−676/−532; data not shown and pGLSP−318/−146, Fig. 5). However, in case of pGL−954/+40, protected band was found at −941/−937 where STAT4, c-Ets-1, c-Ets-2, Elk-1, Pax-5 and p53 could be putative transcription factors. Thus, we could not specify *cis*-elements for pGLSP−676/−532 and pGLSP−318/−146 and EMSA using oligo-DNA was currently unavailable.

## Discussion

It was surprising that pGLSP−676/−1 and pGLSP−318/−1 displayed high luciferase activities in NHEK cells. NHEK cells were cultured in culture media containing 0.15 mM Ca^2+^ to avoid differentiation. Expression of SPINK5 is limited at the very surface of SG, namely SPINK5 expression is limited at the very last step of differentiation just before denucleation [5]. Thus, in NHEK cells without induction of differentiation, no luciferase activity was expected in any of reporter constructs. However, their intensities were only <1/3 of that of pGL−954/+40 containing KLK1 promoter. Expression of KLK1 is observed throughout the SS and SG [5]. It is not surprising that, independent of 0.15 mM Ca^2+^, KLK1 promoter appeared to have an activity in NHEK cells with very high intensity. Therefore, some NHEK seemed to differentiate to the final step in this medium even when Ca^2+^ concentration was maintained at 0.15 mM and growth factors like BPE were included. Growth factors are considered to suppress keratinocyte differentiation [22, 23]. However, neither removal of growth factors alone (low Ca^2+^ medium) nor removal of growth factors and an increase in Ca^2+^ concentration (high Ca^2+^ medium) altered the intensity of luciferase and the movability of EMSA bands, indicating that such stimulation is not suitable for the final differentiation. On the other hand, high cell density reportedly promotes keratinocyte differentiation [23]. It is conceivable that several colonial clones may differentiate further.

TFSEARCH, ALGGEN PROMO and PhysBinder did not find common transcription factors in the activating regions −932/−837, −676/−532 a3d −318/−146. Only SRY (Sex-determining region Y) was common to −932/−837 and −318/−146. However, SRY was encoded on Y chromosome and regulated sex differentiation [24], indicating that this transcription factor was not responsible for skin function. Judging from the results of EMSA, the size of transcription factor binding to −676/−532 and −318/−146 was almost the same. It is possible that the same transcription factor which could not be detected by these programs occupied −932/−837 and −318/−146. Otherwise, the results of DNA footprinting might indicate that more than one protein occupied these sequences.

An epidemiological research with case–control design in Chinese Han population indicated that −206 G > A mutation produced GATA3 binding site in SPINK5 promoter and that the G allele was significantly more common among patients with asthma than among the controls [21]. Existence of GATA3 site might directly increase SPINK5 expression. However, our −206 was G, hence no GATA3 binding site was extracted in neither −676/−532 nor −318/−146. Therefore, GATA3 unlikely affected directly on these sites. Although intensity was still <1/3 of pGL−954/+40, induction of GATA3 in NHEK increased their luciferase activity. On the other hand, transfection of dominant negative GATA3mut suppressed it. The effect of GATA3 was limited and indirect. This may be via transcription factor(s) downstream to GATA3. For example, GATA3 reportedly react with Smads [25] but none of TFSEARCH, ALGGEN PROMO, and PhysBinder found any Smads site in −676/−532 and −318/−146. It seems necessary to explore transcription factors downstream to GATA3 that could regulate these sites. However, unfortunately, we could not yet identify the transcription factor(s) that regulated SPINK5 expression in keratinocytes. Otherwise, inhibition of proliferation and induction of differentiation of keratinocytes by GATA3 [26] might trigger the next step of differentiation of keratinocytes and SPINK5 expression that was regulated by transcription factor(s)/*cis*-element(s) different from GATA3 system. That is, GATA3 is a very important factor for the differentiation of keratinocytes, but GATA3 itself seems to be insufficient for the final differentiation and/or SPINK5 expression.

It is very difficult to obtain sufficient amount of nuclear protein from human skin where SPINK5 expresses. It seems also very difficult to induce final differentiation to keratinocyte culture. High Ca^2+^ stimulation in the present study was insufficient for the final differentiation. Further examination on expression regulation mechanism of −676/−532 and −318/−146 will supply important information related to a key regulator of SPINK5 expression and final differentiation of keratinocytes.
